# Factors Affecting Cell Biomass and Flavonoid Production of *Ficus deltoidea* var. *kunstleri* in Cell Suspension Culture System

**DOI:** 10.1038/s41598-019-46042-w

**Published:** 2019-07-02

**Authors:** Zainol Haida, Ahmad Syahida, Syed Mohd Ariff, Mahmood Maziah, Mansor Hakiman

**Affiliations:** 1 Department of Crop Science, Faculty of Agriculture, Universiti Putra Malaysia, 43400 Serdang, Selangor Malaysia; 2 Department of Biochemistry, Faculty of Biotechnology and Biomolecular Sciences, Universiti Putra Malaysia, 43400 Serdang, Selangor Malaysia

**Keywords:** Plant biotechnology, Secondary metabolism

## Abstract

A study was conducted to establish *in vitro* culture conditions for maximum production of biomass and flavonoid content for *Ficus deltoidea* var. *kunstleri*, locally named as Mas Cotek, known to have a wide variety of potential beneficial attributes for human health. Size of initial inoculum, cell aggregate and initial pH value have been suggested to influent content of biomass and flavonoid for cell suspension culture in several plant species. In the present study, leaf explants were cultured by cell suspension culture procedures in MSB5 basal medium supplemented with predetermined supplements of 30 g/L sucrose, 2.75 g/L gelrite, 2 mg/L picloram and 1 mg/L kinetin with continuous agitation of 120 rpm in a standard laboratory environment. Establishment of cell suspension culture was accomplished by culturing resulting callus in different initial fresh weight of cells (0.10, 0.25, 0.50, 1.0, and 2.0 g/25 mL of media) using similar basal medium. The results showed that the highest production of biomass (0.65 g/25 mL of media) was recorded from an initial inoculum size of 2.0 g/25 mL media, whereas the highest flavonoid (3.3 mg RE/g DW) was found in 0.5 g/25 mL of media. Cell suspension fractions classified according to their sizes (500–750 µm, 250–500 µm, and <250 µm). Large cell aggregate size (500–750 µm) cultured at pH 5.75 produced the highest cell biomass (0.28 g/25 mL media) and flavonoid content (3.3 mg RE/g DW). The study had established the optimum conditions for the production of total antioxidant and flavonoid content using DPPH and FRAP assays in cell suspension culture of *F*. *deltoidea* var. *kunstleri*.

## Introduction

*Ficus deltoidea* Jack, locally known as Mas Cotek, is an evergreen shrub traditionally used in Malaysia as a remedy to treat cardiovascular diseases and diabetes^1^. The plant can grow up to seven meters high with a canopy spread of one to three metres. The leaf is broadly spoon-shaped to ovate and leathery^2^. The species is well-known for its medicinal properties and recognised for many generations for its broad health benefits^3^. Leaf extract has been proven to reduce sugar level, blood pressure and cholesterol, besides being used to treat migraine, remove toxins from the body and reduce the risk of cancer^4^.

Presently, the pharmaceutical industries use numerous plant-based sources for the production of bioactive compounds or secondary metabolites. Several phytochemical compounds have been found in *F*. *deltoidea* extract such as epicatechin, catechin, gallocatechin and epigallocatechin. It also has been reported that polyphenols, flavonoids and tannins were present in *F*. *deltoidea* extract but alkaloids, triterpenes and saponins were absent^5^. Phytochemical compounds such as polyphenols, flavonoids and tannins are the natural source of antioxidant. Antioxidant is important to human in order to neutralize free radicals that present in human body. Free radicals are significantly dangerous molecules as it can cause inflammatory, cardiovascular diseases and cancer. Currently, synthetic antioxidant is available in the market such as butylated hydroxytoluene (BHT) and butylated hydroxyanisole (BHA). However, natural antioxidant is more preferable due to low side effect^6^. Hence, high demand for these plant-based products has led to over-harvesting of wild species and the eventual decrease of many medicinal plant species in nature leading to a definite limitation to their availability^7^. The biotechnological approach of producing plant cells, tissues and organ through *in vitro* cultures has been seen as the alternative system for large scale production of bioactive compounds^8^. Plant cell suspension culture is one recent technique of producing wide variety of secondary metabolites in large quantities in shorter period of time. In such culture system, nutrient components in basal media play important roles in the initiation and production of secondary metabolites by controlling enzyme activities as well as gene expression^9^. The present study was undertaken to investigate the effect of initial size of inoculums, cell aggregate and pH of medium in a cell suspension culture of *Ficus deltoidea* var. *kunstleri* with an aim of establishing the level for optimum production of biomass and flavonoid.

## Materials and Methods

### Plant materials and establishment of cell suspension culture

The leaf explants of *F*. *deltoidea* var. *kunstleri* were obtained from 6-month old plants that were purchased from Department of Agriculture, Serdang, Selangor, Malaysia. The plants were maintained in the glasshouse of Universiti Putra Malaysia. The explants were collected prior to experiment. The explants were washed under running tap water with an addition of a commercial detergent. Leaf surfaces of explants were sterilised using 30% Clorox^®^ with two drops of Tween-20 in a sterilised flask and shaken for 20 minutes. Subsequently, the explants were rinsed three times using sterile distilled water. Leaf explants were excised to 0.5 × 0.5 cm size and cultured onto Murashige and Skoog basal media^10^ fortified with vitamins by Gamborg et al^11^. (MSB5) all under sterile conditions. The basal medium was supplemented with pre-determined supplements of 30 g/L sucrose, 2.75 g/L gelrite, 2 mg/L picloram and 1 mg/L kinetin. The pH of the medium was adjusted to 5.75 prior to autoclave. The cultures were incubated under 16 hours of photoperiod of light and 8 hrs darkness using 2000 lux white fluorescence at laboratory temperature of 25 ± 2 °C. The basal medium used in this experiment was optimised from the previous study by Haida *et al*.^12^.

About 2 ± 0.2 g of two-week old callus was transferred on similar medium formulation without gelrite. The cultures were maintained under continuous agitation at 120 rpm using an orbital shaker for two weeks. The cell suspension cultures were sub-cultured three times for every two weeks using 750 µm sieve size to homogenize and obtain larger amount of cell suspension.

### Effect of initial inoculum size

For effect of initial inoculum size, 12-day old cell suspension with different initial fresh weight of cells (0.10, 0.25, 0.50, 1.0, and 2.0 g/25 mL of media) was cultured. The dry weight and flavonoid content of the cells were measured after 12 days of incubation. The cell suspension was filtered using Whatman No. 1 filter paper and dried in an oven at 50 °C for two days or until the weight remained constant. The initial inoculum size that recorded the highest flavonoid production was used for all subsequent experiments.

### Effect of size of cell aggregate

The 12-day-old cell suspensions were sieved using three different sizes of stainless steel mesh of 750, 500 and 250 µm. Cell suspensions were initially sieved through the biggest stainless steel mesh (750 µm) and washed three times with MSB5 basal medium to avoid damage to the cells due to osmotic stress. The procedures were repeated when using the 500 and 250 µm stainless steel mesh. Cell suspension fractions were classified according to their sizes: 500–750 µm, 250–500 µm, and <250 µm. Each culture vessel contained 0.5 g of cell suspension. The cells were filtered on day 12 and were dried in an oven at 50 °C for two days or until the weight remained constant after which the cells’ dry weight and flavonoid content were measured. The cell aggregate size that produced the highest flavonoid was used in the subsequence experiments.

### Effect of initial pH

In the present experiment, initial pH values of 3.0, 5.0, 5.75, 7.0 and 9.0 were used. pH of 5.75 was used as a control as determined in the previous experiments. The cell suspension used was weighted 0.5 g/25 mL of media with the cell aggregate size of 500–700 µm for each culture vessel. The cells were filtered on day 12 and dried in an oven at 50 °C for two days or until the weight remained constant. The dry weight of the cells and flavonoid content were measured. The pH that produced the highest flavonoid was used in the following experiment.

### Extraction of total antioxidant compounds

The method of extraction of total antioxidant compounds by Wong *et al*.^13^ with minor modifications was adopted. The experiment used 0.5 g each of six-month-old dried leaves and 12-day old cell suspension cultures that had been optimised. The samples were placed in separate 150 mL conical flask covered with aluminium foil. A total volume of 25 mL distilled water was added each flask and placed on an orbital shaker for an hour in the dark at 25 °C. The samples were filtered using Whatman No. 1 filter paper and stored in a freezer maintained at −80 °C.

### DPPH free radical scavenging activity

DPPH (2,2-Diphenyl-1-picrylhydrazyl) assay was conducted following procedures by Wong *et al*.^13^. An amount of 0.1 mM DPPH was prepared in methanol and the initial absorbance was measured at 515 nm using a spectrophotometer until the absorbance remained constant. A total of 40 µL of sample extract was added along with 3 mL of 0.1 mM methanolic DPPH solution. The mixture was incubated for 30 minutes at room temperature. The change in absorbance was measured at 515 nm. The antioxidant activity was based on DPPH free radical scavenging ability of the extract and was expressed as mg trolox equivalent (TE)/g sample.

### Ferric reducing antioxidant potential (frap) assay

The FRAP assay was measured according to method by Wong *et al*.^13^. In essence, 200 µL of sample extract was added to 3 mL of FRAP reagent [300 mM of sodium acetate buffer (pH 3.6), 10 mM of TPTZ [2,4,6-tri(2-pyridyl)-*s*-triazine] solution, and 20 mM of FeCl.6H_2_O] at a ratio of 10:1:1. The mixture was incubated in a water bath at 37 °C for 30 minutes to accelerate the reaction. The increase in absorbance was measured using a spectrophotometer at 593 nm. The ability of the antioxidant to reduce the ferric ions was expressed as mg trolox equivalent (TE)/g sample.

### Total flavonoid extraction

The extraction of flavonoid content was done using method by Marinova *et al*.^14^ with minor modifications. Approximately 0.5 g of six-month old dried leaves and a 12-day-old cell suspension of *F*. *deltoidea* var. *kunstleri* were each homogenised with 50 mL distilled water using pestle and mortar, and subsequently transferred into several covered flasks. Each mixture was centrifuged for 5 mins at 14000 rpm and the supernatant was collected for subsequent procedures.

### Total flavonoid content

The total flavonoid content was determined according to the aluminium chloride colometric method by Marinova *et al*.^14^. Approximately 1 mL sample of extract was added into a test tube with 4 mL of distilled water. Subsequently, 0.3 mL of 5% sodium nitrite was added into the test tube. After 5 minutes, 0.3 mL of 10% aluminium chloride was added and at the sixth minute, and 2 mL of 1 M sodium hydroxide was added into the mixture. Then, 2.4 mL of distilled water was added until the mixture reached the volume of 10 mL. The mixture was mixed thoroughly by a vortex machine and the absorbance was measured at 510 nm. The total flavonoid content was expressed as mg rutin equivalents (RE)/g sample.

### Data analysis

To compare significant differences between treatments, all data were analysed by analysis of variance (ANOVA) of Statistical Analysis Software version 1.4 (SAS). The analysis of means was performed by using Least Significant Difference (LSD) with P < 0.05.

## Results and Discussion

### Effect of initial inoculum size on cell biomass

**Figure 1:**
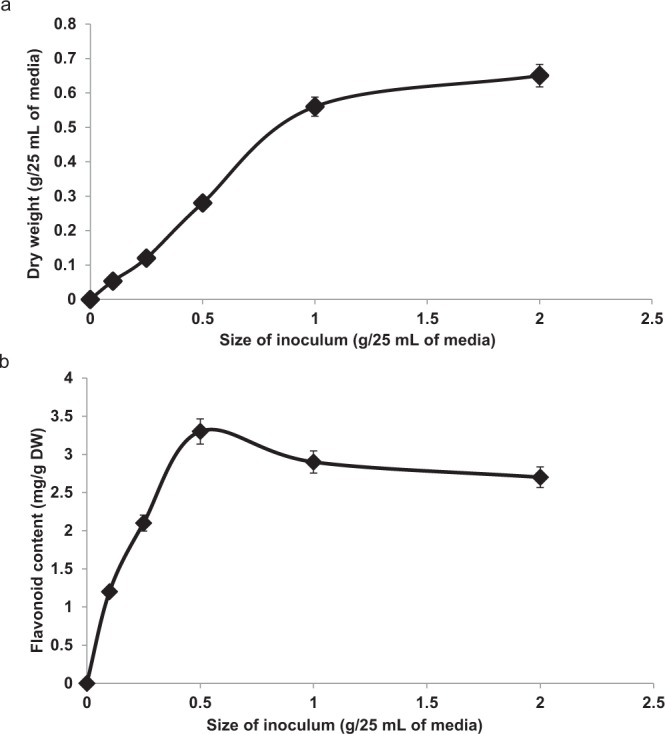
(**a**) Effects of initial inoculum size on biomass of cell suspension of *F*. *deltoidea* var. *kunstleri* (n = 3). Different initial inoculums sizes of cell suspension were used and the dry weight was measured. The figure shows that as the initial inoculums size was increased, the dry weight produced also increased. When the size of inoculums was increased, the cell will divide, thus, will produce more cell and the dry weight will be increased. (**b**) Influence of initial inoculum size on flavonoid production of cell suspension for *F*. *deltoidea* var. *kunstleri* (n = 3). Different inoculums size will directly affect the production of flavonoid produced in the cell suspension. Cell suspension culture is widely used in production of plant secondary metabolites for pharmaceutical industries. Even though as the size of inoculums increased, the dry weight produced also increased, the flavonoid content of the cell was different. The trend produced was sigmoid shape with the size of inoculums 0.5 g/25 mL of media produced the highest. Then, the flavonoid content of the cell was drastically decreased as the size of inoculums increased.

Figure 1a shows that dry weights were affected by the initial inoculum size of cell suspension culture. As the size of the initial inoculum increased, the dry weight increased. The lowest biomass was produced in a 0.1 g/25 mL of media with the dry weight of 0.053 g/25 mL media. This was followed by the initial inoculum size of 0.25, 0.5 and 1.0 g/25 mL of media which produced 0.12, 0.28 and 0.56 g/25 mL of dry weight respectively. The highest dry weight was recorded from treatment using 2.0 g/25 mL of media and initial inoculum size of 0.65 g/25 mL media.

According to a study by Jeong *et al*.^15^, growth and accumulation of ginsenosides in cell suspension culture of ginseng (*Panax ginseng*) produced an optimal biomass of 10.5 g/L, with ginsenoside production of 5.4 mg/g DW and initial inoculum size of 5.0 g/L. Chan *et al*.^16^ conducted a study on different inoculum sizes (0.1, 0.3, 0.5, 1.0 and 1.5 g/30 mL of media) of *Cyperus aromaticus* cell suspension and observed that 0.3 g of initial inoculum size of cell suspension produced the highest fresh and dry weight of biomass. Lower amount of initial inoculum size appeared to perform better than higher inoculum size because increase in the amount of inoculum size, caused nutrient depletion in the media to be even faster^15^. Another study by Saad *et al*.^17^ on *Pogostemon cablin* cell suspension cultures, inoculum size of 1 g/20 mL of media produced the highest cell biomass with dry weight recorded is 0.5 g. From these studies, it indicates that initial inoculum size of the cell is one of the factors that will influence the cell biomass produced.

### Effects of initial inoculum size on flavonoid production

**Figure 2:**
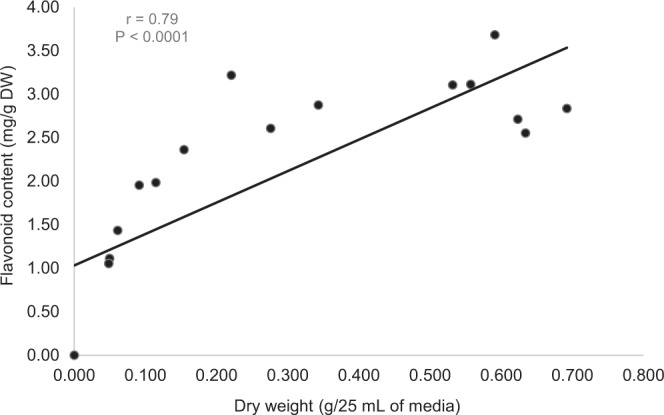
Linear correlation between dry weight and flavonoid content of *F*. *deltoidea* var. *kunstleri* as affected by different initial inoculum size. Solid line indicates a significant linear relationship at P < 0.05. The bullet symbol represent individual replicates, n = 32. There was strong (positive) linear correlation between the dry weight and flavonoid content of the cell suspension.

Dry weight and flavonoid content of cell suspension as affected by different initial inoculum sizes showed a strong linear correlation as shown in Fig. 2. Although the initial inoculum size of 2.0 g/25 mL of media produced the highest biomass that was measured as dry weight, the flavonoid content was not the highest (Fig. 1b). The highest flavonoid production recorded was 3.3 mg RE/g DW from the cell suspension that used the initial inoculum size of 0.5 g/25 mL of media. The initial inoculum size of 0.1 and 0.25 g/25 mL of media produced 1.2 and 2.1 mg RE/g DW of flavonoid. The production of flavonoid showed a declining trend as the initial inoculum size were 1.0 and 2.0 g/25 mL of media which produced 2.9 and 2.7 mg RE/g DW respectively.

A previous study by Li *et al*.^18^ found that inoculums size at 75 g/L produced the highest biomass with 17.16 g/L and the highest chlorogenic acid content of *Lonicera macranthoids* cell suspension cultures. A study conducted by Yang *et al*.^19^ on *Glycyrrhiza inflata* found that 5 g of fresh callus that used as initial inoculum size in 80 mL of MS basal media supplemented with 0.5 mg/L 2,4-D (2,4-dichlorophenoxyacetic acid), 0.5 mg/L NAA (1-napthaleneacetic acid) and 0.5 mg/L BAP (6-benzylaminopurine), produced the highest biomass and flavonoid activity on day 21 with 16.4 g/L DW and 95.7 mg/L.

### Effect of cell aggregate size on cell biomass

**Figure 3:**
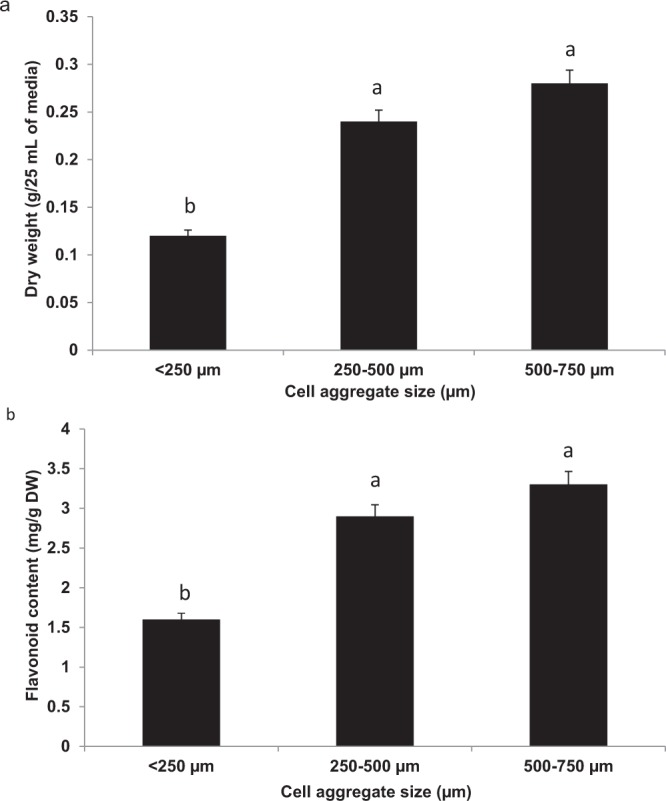
(**a**) Effects of cell aggregate size on the biomass of cell suspension for *F*. *deltoidea* var. *kunstleri* (n = 3). Different size of stainless steel mesh was used to classify cell according to class of sizes. The biggest size of cell aggregate produced the highest dry weight. (**b**) Effects of cell aggregate size on the flavonoid of cell suspension for *F*. deltoidea var. *kunstleri* (n = 3). The flavonoid content produced by the cell was proportionally with the dry weight produced. As the size aggregate of the cells were increased, the flavonoid content of the cells also were increased.

Cell aggregate size plays a major role in the production of high biomass or bioactive compounds in cell suspension culture. In the present study, cell aggregates were classified into three ranges: cell aggregate less than 250 µm, between 250–500 µm, and between 500–700 µm. Firstly, the cell suspension cultures were filtered through 750 µm stainless steel mesh in which the cells with the size that were bigger than 750 µm were discarded. A portion of this filtrate was used to filter through 500 µm stainless steel mesh to obtain the cell aggregate size between 250–500 µm. The stainless steel mesh with the size of 250 µm was used to obtain cell suspension with aggregate size that was less than 250 µm. Figure 3a shows that when the size of cell aggregate increased, the biomasss (dry weight) for the cell suspension of *F*. *deltoidea* var. *kunstleri* also increased. The lowest biomass was produced by the cell aggregate size of 250 µm with 0.12 g/25 mL of media, followed by the cell suspension culture with the aggregate size between 250–500 µm and the highest was produced by 500–750 µm with the biomass of 0.24 and 0.28 g/25 mL media, respectively.

A study on effect of cell aggregate size on biomass was conducted by Miao *et al*.^20^ on *Tripterygium wilfordii*. The cell aggregate was divided into 100, 500, 2000 and 5000 µm. The results found that cell aggregate at size 5000 µm produced the highest cell biomass, followed by cell agrregate at size 2000, 500 and 100 µm. In another study, the influence of cell aggregate size on the biomass production in cell suspension culture of *Beta vulgaris* was carried out by Capataz-Tafur *et al*.^21^. The cell suspension culture was classified into two fractions; <500 µm and >500 µm, obtained by sieving the cell suspension through 500 µm. Our results showed that cell suspension with the fraction of >500 µm produced higher fresh and dry biomass. The finding was in aggrement with a study where larger fraction of cell suspension culture produced higher biomass.

### Effects of cell aggregate size on flavonoid production

**Figure 4:**
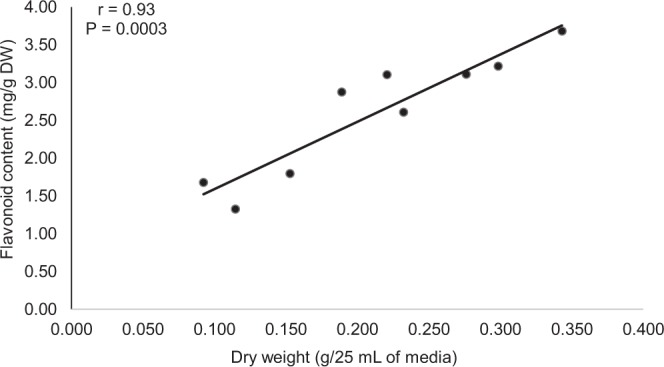
Linear correlation between dry weight and flavonoid content of *F*. *deltoidea* var. *kunstleri* as affected by different cell aggregate size. Solid line indicates a significant linear relationship at P < 0.05. The bullet symbol represent individual replicates, n = 18. There was strong (positive) linear correlation between the dry weight and flavonoid content of the cell suspension.

A correlation analysis was conducted between the dry weight and flavonoid content of cell suspension as affected by cell aggregate size. According to Fig. 4, the dry weight of cell suspension was strongly correlated with flavonoid production of the cell suspension. Referring to Fig. 3b, the highest flavonoid production was detected in cell suspension culture which had cell size of 500–750 µm with 3.3 mg RE/g DW. On the other hand, the lowest flavonoid production in the cell suspension was 1.6 mg RE/g DW for cell aggregate size smaller than 250 µm, followed by 2.9 mg RE/g DW for the cell aggregate size of between 250–500 µm.

A study by Capataz-Tafur *et al*.^21^ on the effects of cell aggregate size on arabinogalactan protein production in cell suspension culture of *Beta vulgaris* was reported. Cell suspension was divided into four fractions; <250 µm, 250–500 µm, 500–1000 µm and  1000 µm, as a result of sieving process through different pore size of 250, 500 and 1000 µm. The results showed that when the size of cells aggregate increased (fraction >1000 µm), the production of arabinogalactan protein also increased. Other than size of cell aggregate, the number of cell aggregate produced in the cell suspension culture can also be the key factor in producing a massive amount of biomass and bioactive compounds.

### Effects of initial ph value on cell biomass

Other factor that can affect biomass and flavonoid production in cell suspension culture is the initial pH value prior to autoclave. The initial pH values play an important role due to the different ability of plants’ tolerance to changes in pH values. The slight difference in pH value can affect the overall production of biomass and bioactive compounds in cell suspension culture. There was no significant difference between pH of the basal medium and cell biomass (Table 1). The highest biomass was produced by cell suspension culture in the control treatment at pH 5.75 with 0.28 g/25 mL of media, followed by the treatment at pH 5.0 and pH 7.0 with the production of biomass of 0.23 and 0.18 g/25 mL of media, respectively. Cell suspension of *F*. *deltoidea* var. *kunstleri* did not survive the initial pH values of pH 3.0 and pH 9.0.

**Table 1:** Effects of initial pH value on biomass and flavonoid production in cell suspension culture of *F*. *deltoidea* var. *kunstleri*. Values are represented as the mean ± standard error (n = 3). na = data not available.

Initial pH Value	Dry Weight (g/25 mL of Media)	Flavonoid Content (mg/g DW)
3.0	na	na
5.0	0.23 ± 0.03^a^	2.50 ± 0.22^b^
5.75 (Control)	0.28 ± 0.04^a^	3.30 ± 0.27^a^
7.0	0.18 ± 0.03^a^	2.75 ± 0.23^ab^
9.0	na	na

A study on cell suspension culture of *Zingiber zerumbet* as affected by pH found that cell suspension culture medium with an initial pH 5.7 exhibited the highest biomass as compared to pH 5.2 and 6.2^22^. In addition, study on *Chlorophytum borivilianum* cell suspension culture, optimum pH value for growth of cell suspension culture was pH 5.86 from a range of pH of 3.86 to 7.86^23^. An experiment by Wu *et al*.^24^ on the effect of initial pH value on the production of flavonoid and phenolic content in adventitious roots of *Echinacea angustifolia* found that the production of biomass, flavonoid, and phenolic content of the species were inhibited when the initial pH was below pH 5.0 and above pH 6.0. Both of the findings were in agreement with the present study.

### Effects of initial ph value on flavonoid production

In the present study, there was no production of biomass in media with pH 3.0 and pH 9.0; hence, both pH value treatments were not able to produce flavonoid (Table 1). The control treatment of pH 5.75 significantly produced the highest flavonoid with 3.30 mg RE/g DW, followed by treatment in media with pH 7.0 and pH 5.0 with flavonoid production of 2.75 and 2.50 mg RE/g DW, respectively.

Tan *et al*.^25^ reported that MS basal media with B5 vitamin, 2 mg/L 2,4-D, 1 mg/L kinetin at pH 5.7 produced the highest flavonoid for *Centella asiatica* L. with four individual flavonoids namely luteolin, apigenin, kaempferol, and rutin. According to Rao and Ravishankar^26^, the preferred initial pH value should be within the range of pH 5.0 to pH 6.0 prior to autoclave and the extreme pH value is avoided as the concentration of hydrogen ions in the basal medium may affect the process of culture development. Slightly acidic condition in the basal medium prior to autoclave is essential in signal transduction process. Acidic basal medium plays a role in forming artificial acidification in the cytoplasm and triggers gene transcription of phenylalanine ammonia lyase, which involves in phenylpropanoid pathway that synthesise flavonoid.

### Comparison of total antioxidant and flavonoid content

The total antioxidant in both DPPH and FRAP assays of the cell suspension cultures were found to be lower than the total antioxidant found in the leaf extracts (Table 2). In DPPH, the free radical scavenging activity of leaf extracts was higher by 3-folds compared to the cell suspension culture with 1.85 and 0.60 mg TE/g, respectively. It was observed that the total antioxidant content using FRAP method showed that the leaf extracts were 6-folds higher than the cell suspension culture with 9.62 and 1.53 mg TE/g, respectively. The total flavonoid content in the leaf extracts was 23.76 mg RE/g, whereas the cell suspension culture was 7-folds lower with 3.30 mg RE/g.

**Table 2:** Comparison of the total antioxidant and flavonoid production in leaf extracts and cell suspension culture of *F*. deltoidea var. *kunstleri*. Values are represented as the mean ± standard error (n = 3).

Biochemical Studies	Leaves (mg/g)	Cell Suspension Culture (mg/g)
Antioxidant DPPH (Trolox equivalent)	1.85 ± 0.2	0.6 ± 0.02
Antioxidant FRAP (Trolox equivalent)	9.62 ± 0.7	1.53 ± 0.05
Total flavonoid (Rutin equivalent)	23.76 ± 1.2	3.30 ± 0.27

In another study, it was found that the total phenol, antioxidant content, and flavonoids of root suspension cultures of *Hypericum perforatum* increased in response to the different concentrations of sucrose treatment using the DPPH method^27^. The production of flavonoids in the cell suspension culture of *Gingko biloba* with the addition of 80 µM ABA (abscisic acid) and fungal endophytes in the light for 24 days produced flavonoids which was two times higher compared to the cell suspension culture with 80 µM ABA, without the addition of fungal endophytes with 2.5 and 1.5% DW^28^, respectively.

Based on the results obtained in Table 2, the production of antioxidant in cell suspension culture was inferior to leaves. However, the present study was at the early stage of the cell suspension culture of *F*. *deltoidea* to establish the best culture conditions to produce the secondary metabolites. The production of secondary metabolites of cell suspension culture was lower than leaves. In addition, specific flavonoid such as rutin was detected in the leaves with 12.83 µg/g DW and cell suspension culture with 3.13 µg/g DW (data not shown). Other than that, naringin also was detected in the leaves sample, however, did not detected in the cell suspension culture of *F*. *deltoidea*. It suggests that cell suspension culture of *F*. *deltoidea* was able to produce secondary metabolites. In this study, the antioxidant of the cell suspension cultures was compared with the leaves. This is because leaves are the main part of *F*. *deltoidea* that contain high antioxidant. The production of secondary metabolites of *F*. *deltoidea* could be maximized by using elicitor or precursor feeding. An elicitor is a substance used in low concentration to initiate or increase the biosynthesis of specific compounds in living cell system^29^. The most common elicitor use in cell suspension culture is jasmonic acid and methyl jasmonate. In cell suspension culture of *Panax ginseng*, methyl jasmonate successfully increased the production of ginsenoside^30^. Similar finding on methyl jasmonate used as elicitor in the production of secondary metabolites include cell suspension culture of *Taxus chinensis*^31^ and *Panax notoginseng*^32^. Others examples of elicitor are salicylic acid, nickel sulphate and ammonium metavanadate. Besides elicitor, one of the novel techniques in improvement of secondary metabolites production in cell suspension culture is precursor feeding. However, precursors are toxic to culture if not used at the appropriate concentration and stage of culture^33^.

## Conclusion

From the present study, different factors including initial inoculums size, cell aggregate and initial pH value were used to increase biomass and flavonoid content of cell suspension culture of *Ficus deltoidea* var. *kunstleri*. For the biomass production of *F*. *deltoidea* var. *kunstleri*, initial inoculum at 2.0 g/25 mL media, cell aggregate at size 500–750 µm and initial pH at 5.75 were found to be the most suitable. In term of flavonoid production, initial inoculum at 0.5 g/25 mL media, cell aggregate at size 500–750 µm and initial pH at 5.75 can maximized the production of flavonoid in cell suspension culture of *F*. *deltoidea* var. *kunstleri*. The production of plant secondary metabolites through plant tissue culture technique especially via cell suspension culture and hairy root culture are more preferable because the plant secondary metabolites production can be controlled by the system or by application of various types of phytohormone. The findings may contribute to the body of knowledge in the pharmaceutical industries and fulfil the demand for the compounds in a large amount.
